# Supplementary figures and images for: Recording γ-secretase activity in living mouse brains
Source: eLife. 2024 Oct 3;13:RP96848. doi: 10.7554/eLife.96848 (PMC11449482; doi:10.7554/eLife.96848)

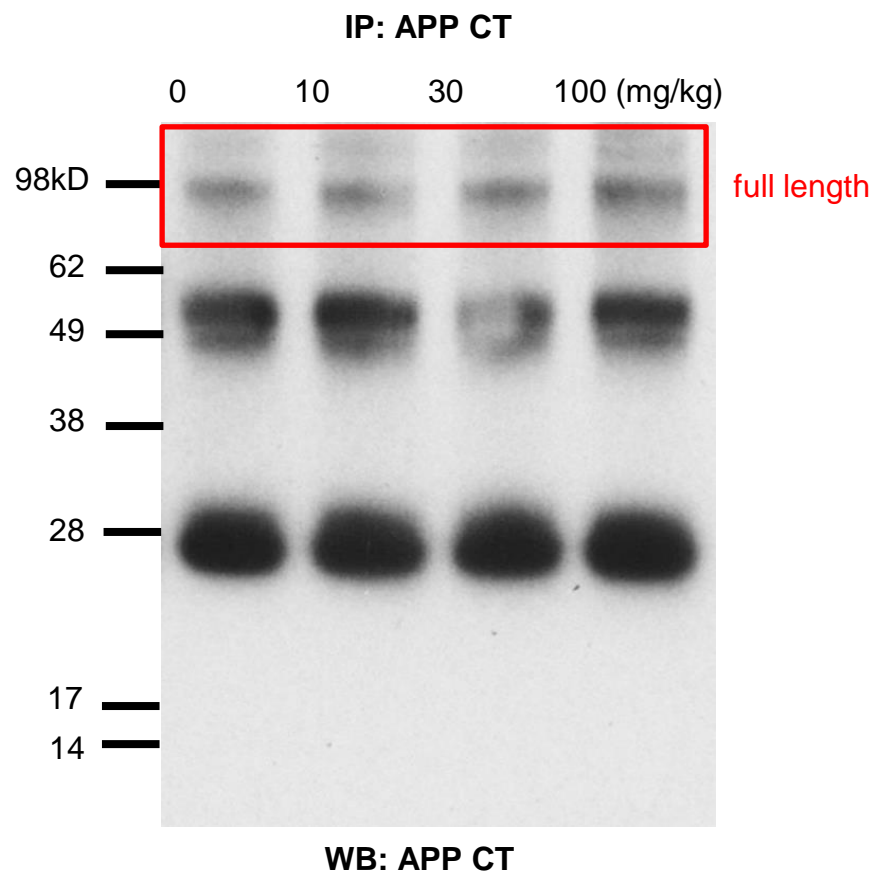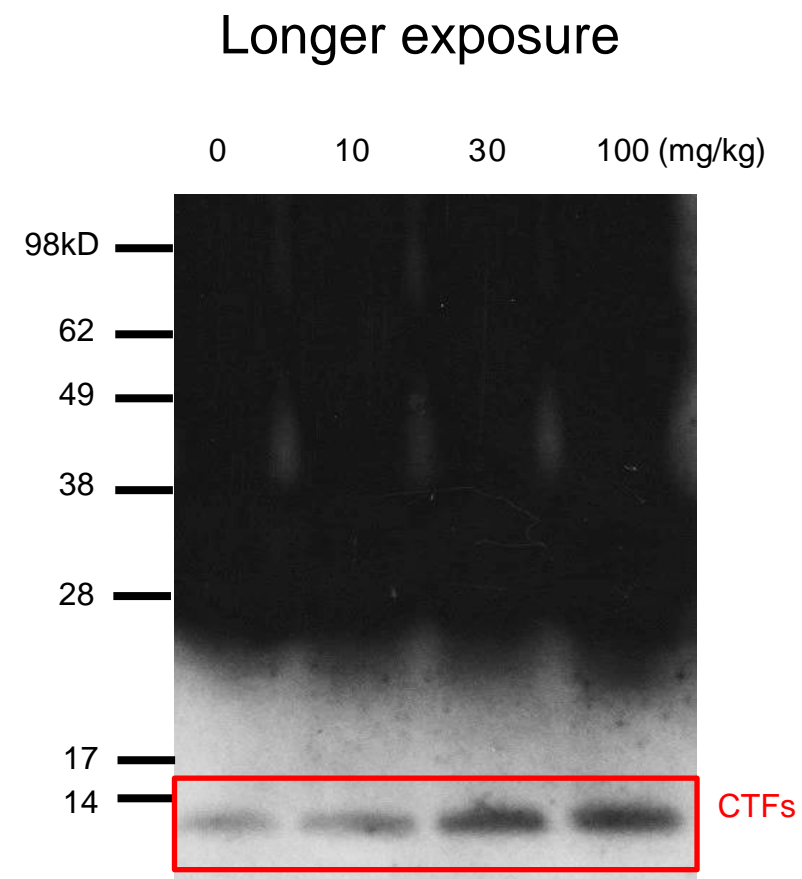

Supplement: Figure 2—figure supplement 2—source data 1. [file elife-96848-fig2-figsupp2-data1.zip › Figure 2-figure supplement 2-source data 1/Uncropped and labelled gels for Figure 2-figure supplement 2.pdf]

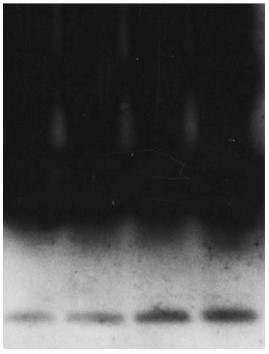

Supplement: Figure 2—figure supplement 2—source data 2. [file elife-96848-fig2-figsupp2-data2.zip › Figure 2 - figure supplement 2 - source data 2 (long expo).JPG]

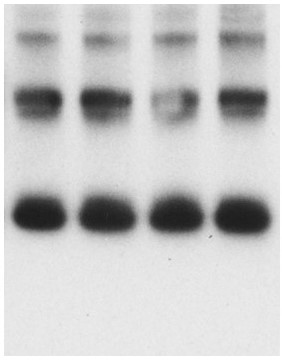

Supplement: Figure 2—figure supplement 2—source data 2. [file elife-96848-fig2-figsupp2-data2.zip › Figure 2 - figure supplement 2 - source data 2.JPG]
